# Diagnostic accuracy of cerebrospinal fluid lactate in confirmed cases of acute bacterial meningitis in children

**DOI:** 10.12669/pjms.36.7.1682

**Published:** 2020

**Authors:** Hina Nasir, Muhammad Faheem Afzal, Muhammad Haroon Hamid, Akmal Laeeq

**Affiliations:** 1 Dr. Hina Nasir, MBBS. Department of Pediatrics, King Edward Medical University, Lahore, Pakistan; 2 Dr. Muhammad Faheem Afzal, FCPS, MHPE. Department of Pediatrics, King Edward Medical University, Lahore, Pakistan; 3 Muhammad Haroon Hamid, FCPS. Department of Pediatrics, King Edward Medical University, Lahore, Pakistan; 4 Akmal Laeeq, FCPS. Department of Pediatrics, King Edward Medical University, Lahore, Pakistan

**Keywords:** Bacterial meningitis, Cerebrospinal fluid, Diagnostic biomarker, Lactate level

## Abstract

**Objective:**

To determine the diagnostic accuracy of cerebrospinal fluid lactate level in confirmed cases of acute bacterial meningitis in children

**Methods:**

This cross sectional study was conducted in the Department of Paediatrics, King Edward Medical University/ Mayo Hospital, Lahore from January to December 2018. A total of 250 children, between two months - 12 years of age, of both the genders, with suspected acute bacterial meningitis were included by non-probability consecutive sampling. Each child was subjected to lumbar puncture for biochemistry, cytology, culture, and lactate level. CSF lactate level of 1.1-2.4 mmol/L was taken as normal, and >2.4 mmol/L was taken as cut off for acute bacterial meningitis. All collected data was entered and analyzed in SPSS version 22. A 2 x 2 table was made to calculate diagnostic accuracy, sensitivity, specificity, positive and negative predictive value for CSF Lactate.

**Results:**

The sensitivity, specificity, positive predictive value, negative predictive value and diagnostic accuracy of CSF lactate taking CSF culture as gold standard was 100%, 60.61%, 17.27%, 100% and 63.6% respectively, with kappa of 0.19 and p value of 0.000.

**Conclusion:**

At a cut off value of 2.4 mmol/L, cerebrospinal fluid lactate level has a high diagnostic accuracy for acute bacterial meningitis.

## INTRODUCTION

Acute bacterial meningitis (ABM), inflammatory disease of the leptomeninges causing characteristic changes in the cerebrospinal fluid (CSF), is one of the most common central nervous system infections in Pediatric population. *Haemophilus influenzae, Streptococcus pneumoniae and Neisseria meningitidis* are the most common causative organisms in children more than two months of age.^1^ The median incidence for bacterial meningitis is 34.0 per 100,000 child-years globally, with a median case-fatality rate of 14.4%.^2^ Bacteria and enteroviruses are the main cause of acute community-acquired meningitis. Bacterial meningitis is associated with high morbidity and mortality.^3^ Prompt treatment with appropriate antibiotics is essential to optimize outcomes. Early diagnosis is therefore crucial for selecting patients who need antibiotics. On the other hand, the course of viral meningitis is in general benign and there is usually no specific treatment.^4^

CSF lactate has been recently advocated as a useful diagnostic test. This may help not only in the early diagnosis of ABM, but also differentiating ABM from aseptic meningitis.^5^ CSF lactate is the enzyme that is produced by anaerobic metabolism and its level increases in any condition which leads to decrease in oxygen supply to the brain as in bacterial meningitis. One important characteristic is that this does not depend on the blood lactate level.^6^ Filho et al.,^7^ Chen et al.^8^, Barros et al.^9^, Giulieri et al.^10^ and Cunha et al.^11^ found that CSF lactate may be a good biomarker that can differentiate bacterial meningitis from aseptic meningitis. Meta-analyses by Sakushima et al.^12^ and Huy et al.^13^ have reported excellent predictive power for lactate and also showed reduced diagnostic power of lactate in patients who had previously received antimicrobial drugs.

Generally, most patients with clinical suspicion of acute meningitis are treated with broad spectrum antibiotics targeting bacterial meningitis leading to increase antibiotic resistance and adverse effects in our setup. In past, studies have been conducted to determine the role of CSF lactate for differentiating acute bacterial meningitis from aseptic meningitis. The objective of this study was to determine the diagnostic accuracy of cerebrospinal fluid lactate level in children with bacterial meningitis taking CSF culture as gold standard.

## METHODS

This cross sectional study was conducted in the Department of Paediatrics, King Edward Medical University/Mayo Hospital Lahore from January to December 2018. The study was approved by the institutional review board (Ref. No. 129/RC/KEMU, Dated: 10-02-2017) and was funded by research grant of King Edward Medical University, Lahore. Informed consent was taken from patient’s parents/guardian. A total of 250 children of both the genders, between 2months-12 years of age, with clinical diagnosis of suspected meningitis presenting within 72 hours of symptoms were included by non-probability consecutive sampling. (The sample size is calculated using sensitivity and specificity of CSF Lactate in bacterial meningitis as 95.0%^6^ and 93.6%^6^ with 3% margin of error). A child with acute onset of fever (usually > 38.5 °C rectal or 38.0°C axillary), headache and one of the following signs: neck stiffness, altered consciousness (GCS <13/15) or other meningeal signs (bulging fontanelle in <1 year old) was labeled as suspected case of acute bacterial meningitis while labeled as confirmed by a positive CSF culture.^14^ Patients who received antibiotics within 48 hours prior to presenting to the hospital, patients with tuberculosis meningitis (having clinical history of symptoms > 2weeks and CSF picture of predominant lymphocytes), and patients on immunosuppressive therapy (as evident from clinical record) were excluded from the study. Demographic and clinical data of every patient were obtained. Each child was subjected to lumbar puncture. Three collection tubes with patient’s name, date and time of specimen collection, and unique identification number was prepared before taking samples, pre-procedure vitals was recorded. Proper biosafety guidelines were followed while taking samples. About three ml of CSF sample was taken (1ml in each tube for biochemistry, gram staining and culture, and lactate level) and was sent to the Paediatrics Microbiology and biochemistry laboratory within one hour of sample collection for CSF cytology, biochemistry and microbiology. After appropriate centrifugation, smear was prepared using the CSF sediment should and was visualized under microscope. The CSF samples were centrifuged at 4000 rpm for 5min and were inoculated to 5% sheep blood agar, EMB agar and chocolate agar. Samples inoculated to the media were stored in the incubator at 37 °C for 24 and 48h. At the end of the incubation period, the plates were assessed through the conventional method. Identification of the plates on which growth was observed was carried out. CSF lactate level was measured using standard enzymatic methods. Reports were collected within standard reporting time for respective tests. As per manufacturer guide, CSF lactate level of 1.1-2.4 mmol/L was taken as normal, and >2.4 mmol/L was taken as cut off for acute bacterial meningitis. Each child was treated according to the individual merit. All information was recorded on a structured questionnaire.

All collected data was entered and analyzed in SPSS version 22. Quantitative data like age was presented in form of mean ± S.D. The qualitative data like gender, bacterial meningitis on CSF Lactate and CSF Culture was presented as frequency and percentage. A 2 x 2 table was made to calculate diagnostic accuracy, sensitivity, specificity, positive and negative predictive value for CSF Lactate taking CSF Culture as gold standard.

## RESULTS

The mean age of cases was 16.23 ± 15.86 months. There were 137(54.8%) male and 113(45.2%) female cases. Mean ± SD of CSF lactate was 2.88±0.24 mmol/L. There were 19 (7.6%) positive cases of acute bacterial meningitis on CSF culture. Among culture positive cases, 12 (4.8%) cases were of *Streptococcus pneumoniae* while 7(2.8%) cases were of *Neisseria meningitides*. The sensitivity, specificity, positive predictive value, negative predictive value and diagnostic accuracy of CSF lactate taking CSF culture as gold standard was 100% (95% confidence interval (CI) 83.18-100%), 60.61% (95% CI 54.18-66.68%), 17.27% (95% CI 11.35-25.41%), 100% (95% CI 97.33-100%), and 63.6% (95% CI 57.47-169.32%) respectively. Agreement of findings of culture & CSF lactate level was 0.19, that is statistically significant (p=0.000) (Table-I). The sensitivity, specificity, positive predictive value, negative predictive value and diagnostic accuracy of CSF lactate taking gram staining was 85.71%(95% CI 77.08-91.46%), 79.87%(95% CI 72.97-85.37%), 70.91%(95% CI 61.83-78.58%), 90.71%(95% CI 84.76-94.49%), and 82%(95% CI 76.76-86.27%) respectively. Agreement of findings of staining & CSF lactate level was 0.68, that is statistically significant (p=0.000) (Table-II).

**Table-I:** Diagnostic accuracy of CSF lactate in acute bacterial meningitis taking CSF culture as gold standard.

		Bacterial meningitis on Culture		Total

		Positive	Negative
Bacterial meningitis on CSF lactate	Positive	19	91	110
	Negative	0	140	140

Total	19	231	250	

Sensitivity: 100%, Specificity: 60.61%, PPV: 17.27%, NPV:100% kappa:0.19, p=0.000

**Table-II:** Diagnostic accuracy of CSF lactate in acute bacterial meningitis taking CSF gram staining.

		Bacterial meningitis on Gram staining		Total

		Positive	Negative	
*Bacterial meningitis on CSF lactate*	*Positive*	78	32	110
	*Negative*	13	127	140

*Total*	91	159	250

Sensitivity: 85.71%, Specificity: 79.87%, PPV: 70.91%, NPV: 82%, kappa: 0.68, p=0.000

## DISCUSSION

In current study, sensitivity, specificity, positive predictive value, negative predictive value and diagnostic accuracy of CSF lactate taking CSF culture as gold standard was 100%, 60.61%, 17.27%, 100% and 63.6% respectively. We found our results comparable with previous studies. Filho EM et al.^7^, reported sensitivity of 95%, specificity of 94% and negative predictive value of CSF lactate as 99.3% for bacterial meningitis. Similarly, Chen et al.^8^ found that CSF lactate had diagnostic sensitivity of 94.7%. In a meta-analysis, Sakushima et al.^12^ found that pooled test characteristics of CSF lactate at the level of 3.8 mmol/L were, sensitivity 0.93 (95%CI: 0.89-0.96), specificity 0.96 (95% CI: 0.93-0.98), likelihood ratio positive 22.9 (95%CI: 12.6-41.9), likelihood ratio negative 0.07 (95%CI: 0.05-0.12), and diagnostic odds ratio 313 (95%CI: 141-698). Huy et al.^13^ compared CSF biochemistry with lactate and reported higher diagnostic accuracy of the CSF lactate. Authors from a study conducted in adult population concluded that CSF lactate level was significantly high in bacterial than viral meningitis.^15^ Nazir et al.^5^ from India showed that while at a cut-off value of 3 mmol/L, CSF lactate has high diagnostic accuracy for bacterial meningitis, mean levels in viral meningitis remain essentially below 2 mmol/L. One meta-analysis conducted by Xiao et al.^16^ also concluded the same results. Giulieri et al.^10^ also suggested that CSF lactate had the highest accuracy for discriminating bacterial from viral meningitis, with a cutoff set at 3.5 mmol/l and should be included in the initial diagnostic workup of this condition. Julián-Jiménez et al.^1^, Domingues et al.^17^ and Buch et al.^18^ also reported that CSF lactate was more accurate than other CSF markers to identify bacterial meningitis. Pires et al.^19^ found lactate as the best single CSF marker of bacterial meningitis with high sensitivity and specificity of CSF lactate in a pediatric population. Our findings confirm the findings of previous studies showing CSF lactate as the reliable CSF marker of bacterial meningitis.

We believe that this is among the first studies from Punjab highlighting the importance of CSF lactate in diagnosis of acute bacterial meningitis. We could not find out local references to compare our results. We used the kit which has cut off range of 2.4 mmol/L, so to take care for the cut off, manufacturer guide needs to be followed. Viral studies were not performed due to limitation of resources.

**Fig.1:**
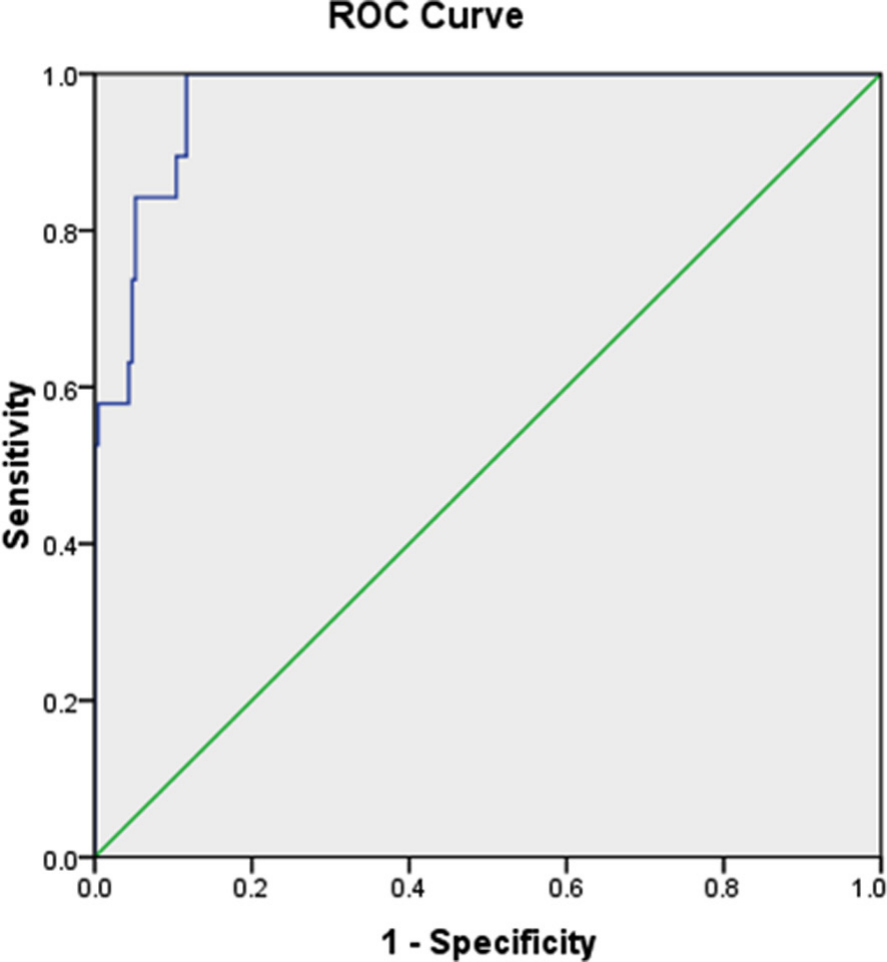
Receiver operating curve (ROC).

## CONCLUSION

At a cut off value of 2.4 mmol/L, cerebrospinal fluid lactate level has a high diagnostic accuracy for acute bacterial meningitis. So this biomarker can provide rapid and reliable diagnostic information for early diagnosis of acute bacterial meningitis. However, CSF lactate determination should not replace the conventional gold standard tests for meningitis.

### Authors’ Contribution:

**MFA** conceived, designed and did statistical analysis & editing of manuscript, is responsible for integrity of research. **MFA, HN & MHH** did data collection and manuscript writing. **AL** did review and final approval of manuscript.

## References

[1] Julián-Jiménez A, Morales-Casado M (2019). Usefulness of blood and cerebrospinal fluid laboratory testing to predict bacterial meningitis in the emergency department. Neurología.

[2] Luksic I, Mulic R, Falconer R, Orban M, Sidhu S, Rudan I (2013). Estimating global and regional morbidity from acute bacterial meningitis in children:assessment of the evidence. Croat Med J.

[3] van Ettekoven CN, van de Beek D, Brouwer MC (2017). Update on community-acquired bacterial meningitis:guidance and challenges. Clin Microbiol Infect.

[4] McGill F, Griffiths MJ, Solomon T (2017). Viral meningitis:current issues in diagnosis and treatment. Curr Opin Infect Dis.

[5] Nazir M, Wani WA, Malik MA, Mir MR, Ashraf Y, Kawoosa K (2018). Cerebrospinal fluid lactate:a differential biomarker for bacterial and viral meningitis in children. J Pediatr.

[6] Ramljak S, Schmitz M, Zafar S, Wrede A, Schenkel S, Asif AR (2015). Cellular prion protein directly interacts with and enhances lactate dehydrogenase expression under hypoxic conditions. Exp Neurol.

[7] Filho EM, Horita SM, Gilio AE, Nigrovic LE (2014). Cerebrospinal fluid lactate level as a diagnostic biomarker for bacterial meningitis in children. Int J Emerg Med.

[8] Chen Z, Wang Y, Zeng A, Chen L, Wu R, Chen B (2012). The clinical diagnostic significance of cerebrospinal fluid d-lactate for bacterial meningitis. Clin Chim Acta.

[9] Barros DP, Peres FGB, de Moura LFB, Carlos S (2019). Performance of lactate in discriminating bacterial meningitis from enteroviral meningitis. Rev Inst Med Trop S Paulo.

[10] Giulieri S, Chapuis-Taillard C, Jaton K, Cometta A, Chuard C, Hugli O (2015). CSF lactate for accurate diagnosis of community-acquired bacterial meningitis. Eur J Clin Microbiol Infect Dis.

[11] Cunha BA (2012). Cerebrospinal fluid (CSF) lactic acid levels:a rapid and reliable way to differentiate viral from bacterial meningitis or concurrent viral/bacterial meningitis. J Clin Microbiol.

[12] Sakushima K, Hayashino Y, Kawaguchi T, Jackson JL, Fukuhara S (2011). Diagnostic accuracy of cerebrospinal fluid lactate for differentiating bacterial meningitis from aseptic meningitis:a meta-analysis. J Infec.

[13] Huy NT, Thao NT, Diep DT, Kikuchi M, Zamora J, Hirayama K (2010). Cerebrospinal fluid lactate concentration to distinguish bacterial from aseptic meningitis:a systemic review and meta-analysis. Crit Care.

[14] World Health Organization (2014). Bacterial meningitis (including Haemophilus influenzae type b (Hib), Neisseria meningitidis, and Streptococcus pneumoniae) [Online].

[15] Abro AH, Abdou AS, Ustadi AM, Saleh AA, Younis J, Doleh WF (2009). CSF lactate level:a useful diagnostic tool to differentiate acute bacterial and viral meningitis. J Pak Med Assoc.

[16] Xiao X, Zhang Y, Zhang L, Kang P, Ji N (2016). The diagnostic value of cerebrospinal fluid lactate for post-neurosurgical bacterial meningitis:a meta-analysis. BMC Infect Dis.

[17] Domingues RB, Fernandes GBP, Leite FBVM, Carlos S (2019). Performance of lactate in discriminating bacterial meningitis from enteroviral meningitis. Rev Inst Trop Med São Paulo.

[18] Buch K, Bodilsen J, Knudsen A, Larsen L, Helweg-Larsen J, Storgaard M (2018). Cerebrospinal fluid lactate as a marker to differentiate between community-acquired acute bacterial meningitis and aseptic meningitis/encephalitis in adults:A Danish prospective observational cohort study. Infect Dis (Lond).

[19] Pires FR, Franco AC, Gilio AE, Troster EJ (2017). Use of score and cerebrospinal fluid lactate dosage in the differential diagnosis of bacterial and aseptic meningitis. Rev Paul Pediatr.

